# Uric acid is associated with cognitive impairment in the elderly patients receiving maintenance hemodialysis—A two‐center study

**DOI:** 10.1002/brb3.1542

**Published:** 2020-01-27

**Authors:** Jing Zhang, Lijuan Tang, Jun Hu, Yuwei Wang, Yuzhen Xu

**Affiliations:** ^1^ Department of Nephrology Wannan Medical College Affiliated Yijishan Hospital Wuhu China; ^2^ Department of Nephrology Wuhu Second People's Hospital Wuhu China; ^3^ Blood Purification Center Wannan Medical College Affiliated Yijishan Hospital Wuhu China; ^4^ Department of Neurology Shanghai Tenth People's Hospital Tongji University Shanghai China; ^5^ Department of Neurology Taian City Central Hospital Shandong First Medical University & Shandong Academy of Medical Sciences Taian China

**Keywords:** cognitive impairment, end‐stage renal disease, maintenance hemodialysis, uric acid

## Abstract

**Background:**

Elevated serum uric acid (SUA) has been associated with vascular cognitive impairment (CI) in the elderly. However, its relationship with cognitive function in the elderly patients receiving maintenance hemodialysis (MHD) has not yet been elucidated.

**Objective:**

The cognitive impairment is prevalent in MHD patients. Various insults may contribute to cognitive impairment in patients with MHD. The aim of this study was to investigate the relationship between SUA and CI in the elderly patients receiving MHD.

**Methods:**

A total of 180 elderly MHD subjects from two hospitals were enrolled in our study. Cognitive function domains were evaluated by MMSE at the beginning of the trial. Demographic and clinical characteristics were collected and recorded.

**Results:**

The subjects were stratified into quartiles according to SUA level. Demographic and clinical characteristics such as age, gender, smoking habit, education year, blood pressure (BP), hemoglobin, albumin, blood glucose (BG), total cholesterol (TC), triglycerides (TG), high‐density lipoprotein cholesterol (HDL), low‐density lipoprotein cholesterol (LDL), blood urea nitrogen (BUN), and serum creatinine (Scr) did not differ dramatically among groups (*p* > .05). The Q1 group showed the highest MMSE scores, and the Q4 group showed the lowest MMSE sores (*p* < .05). There was a negative correlation between SUA and MMSE scores (*r* = −.307, *p* = .014), and this correlation was independent of demographic and clinical characteristics.

**Conclusions:**

Elevated SUA maybe contributes to CI in the elderly MHD patients. SUA level is independent risk for the CI in the elderly MHD patients.

## Graphical abstract

It was reported elevated serum uric acid (SUA) has been associated with vascular cognitive impairment (CI) in the elderly. However, its relationship with cognitive function in the elderly patients receiving hemodialysis (HD) has not been elucidated. Herein, we enrolled 180 elderly HD subjects from two hospitals. Their cognitive function domains were evaluated using MMSE scores at the beginning of the trial, and their demographic and clinical characteristics were collected. We found that the elevated SUA maybe contributes to cognitive impairment in the elderly HD patients.

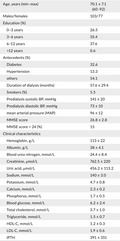

## 1 INTRODUCTION

It was reported that patients at all stages of chronic kidney disease (CKD) are at a higher risk of developing cognitive impairment (CI) (Bugnicourt, Godefroy, Chillon, Choukroun, & Massy, 2013) compared to the general population. The prevalence of CI in hemodialysis (HD) patients is higher (Kurella Tamura et al., 2010). Therefore, CI is likely to become a more and more serious problem (Pereira, Weiner, Scott, & Sarnak, 2005). Most importantly, CI is an independent predictor of all‐cause mortality in maintenance hemodialysis (MHD) patients. Long‐term HD also resulted in a significant almost threefold higher hazard for all‐cause mortality (Angermann et al., 2018). In spite of these, it is not paid enough attention by nephrologists yet (Kurella Tamura & Yaffe, 2011), which should raise awareness about the associated factor causing CI in this population.

Cognitive decline in patients with MHD may be caused by various factors such as cerebrovascular disease, anemia, hypertension, diabetes, and malnutrition (Pereira et al., 2005). Besides these, uremic toxins including higher serum uric acid (SUA) (Bugnicourt et al., 2013) are considered as a risk factor for CI in end‐stage renal disease (ESRD) patients. However, limited studies have investigated the level of SUA on cognitive function in the elderly patients receiving MHD. Thus, we aim to investigate the relationship of SUA level and CI among the elderly patients receiving MHD.

## 2 PATIENTS AND METHODS

### 2.1 Patients

A group of 180 ESRD patients (103 males and 77 females) receiving MHD more than 6 months were recruited from Wannan Medical College affiliated Yijishan Hospital and Wuhu Second People's Hospital. Patients with dementia, infection, uric acid‐lowering therapy, or ongoing serious illnesses such as severe anemia, malignancy, or cerebrovascular disease during the 3 months before the beginning of the study were excluded. All the patients' individual information including sociodemographic data (age, gender, and years of education), primary cause of kidney failure (hypertension, diabetes, and others), and clinical associated data was collected and recorded. The study was approved by the Ethics Committee of Wannan Medical College affiliated Yijishan Hospital and Wuhu Second People's Hospital. The written patients' approval consents were signed by the patients and/or their guardians.

### 2.2 Hemodialysis method

All the patients underwent hemodialysis routinely through internal arteriovenous fistula for three times per week and 4 hrs each time, using the “Gambro” hemodialysis machine (Gambro Lundia AB) and “Gambro” Polyflux L capillary dialyzers (Gambro Dialysatoren GmbH) and bicarbonate dialysate (Na+:138 mmol/L; K+:2.0 mmol/L; Ca2+:1.50 mmol/L; Mg2+:0.5 mmol/L; Cl−:109.5 mmol/L; and HCO3−:32 mmol/L). They were treated with low molecular heparin for anticoagulation (Hangzhou Jiuyuan Gene Engineering Co., Ltd.), according to body weight. The blood flow rate ranged from 180 to 250 ml/min, and dialysis flow rate was maintained at 500 ml/min. All patients were supplied with recombinant human erythropoietin (rhEPO, Beijing Four Rings Biopharmaceutical Co., Ltd.) and L‐carnitine (Reyoung Pharmaceutical Co., Ltd.) after each session of HD.

### 2.3 Cognitive function evaluation

All the subjects were conducted with a Chinese translation version of the MMSE test at the beginning of the study. The MMSE test is the most widely used screening cognitive state of the patients for the clinician (Folstein, Folstein, & McHugh, 1975) and also applied in the research of nephrology field. It includes orientation, memory, attention, executive function, language ability, and visuospatial ability. The maximum score is 30, and cognitive impairment is defined as a score of less than 24 by the same clinical neuropsychologist. The lower is score, the poorer is cognitive ability.

### 2.4 Blood sampling and analysis

Blood samples were collected from antecubital vein of the MHD patients, which were fasted for at least 8 hrs. The serum samples were immediately separated after centrifugation for 10 min at 1200*g* and subjected to the measurement of biochemical parameters including liver function tests (albumin), kidney function tests (uric acid, blood urea nitrogen, and creatinine), blood glucose level, lipids profiles, and electrolyte (potassium, sodium, calcium, and phosphorus) by Hitachi 917 (Hitachi Corp., Roche‐Diagnostic). The intact parathyroid hormone (iPTH) was tested using ADVIA Centaur^®^ XP (Siemens Healthcare).

### 2.5 Data analysis

All the data were analyzed by SPSS 22.0 statistical software. Quantitative variables were expressed as mean ± *SD*, median (min–max), or percentage. The differences between the groups were analyzed by one‐way ANOVA following with Tukey post hoc test. We also performed correlation and regression analyses between demographic (age, gender, education years, etc.) and clinical (hemoglobin, albumin, SUA, etc.) characteristics and MMSE scores. *p* < .05 was considered statistically significant.

## 3 RESULTS

In total, 185 subjects enrolled this study. Of these, five were excluded because they took drugs that could interfere with SUA level during study. As a result, a total of 180 patients comprising 103 (57.2%) males and 77 (42.8%) females were evaluated in this study. The mean age of the participants was 70.1 ± 7.1 years old, and the range was from 60 to 92 years old. About 32.6% patients had diabetes as the primary cause of kidney failure, and 13.3% patients had antecedents of arterial hypertension. About 15% patients showed cognitive impairment (MMSE score < 24). In our study, the average score of MMSE was 26.8 ± 2.8. MMSE scores ranged between 18 and 30 in all subjects. Demographic and clinical characteristics and MMSE scores of the participants grouped by SUA quartiles are presented in Tables 1 and 2. There was no significance in age, gender, years of education, smoking habit, duration of dialysis, SBP, DBP, and clinical characteristics except MMSE scores among the SUA quartiles. However, the higher is SUA quartiles, the worse may be cognitive ability.

**Table 1:** Demographic and clinical characteristics and MMSE scores of MHD patients (*n* = 180)

Age, years (min–max)	70.1 ± 7.1 (60–92)
Males/females	103/77
Education (%)	
0–3 years	26.5
3–6 years	35.4
6–12 years	37.6
>12 years	0.6
Antecedents (%)	
Diabetes	32.6
Hypertension	13.3
others	54.1
Duration of dialysis (months)	57.6 ± 29.4
Smokers (%)	5.5
Predialysis systolic BP, mmHg	141 ± 20
Predialysis diastolic BP, mmHg	73 ± 10
mean arterial pressure (MAP)	96 ± 12
MMSE score	26.8 ± 2.8
MMSE score < 24 (%)	15
Clinical characteristics	
Hemoglobin, g/L	113 ± 22
Albumin, g/L	38 ± 4.1
Blood urea nitrogen, mmol/L	24.4 ± 8.4
Creatinine, μmol/L	762.5 ± 220
Uric acid, μmol/L	456.2 ± 113.2
Sodium, mmol/L	140 ± 3.0
Potassium, mmol/L	4.7 ± 0.8
Calcium, mmol/L	2.3 ± 0.2
Phosphorus, mmol/L	1.7 ± 0.5
Blood glucose, mmol/L	6.2 ± 2.4
Total cholesterol, mmol/L	3.7 ± 1.0
Triglyceride, mmol/L	1.5 ± 0.7
HDL‐C, mmol/L	1.2 ± 0.3
LDL‐C, mmol/L	1.9 ± 0.6
iPTH	391 ± 351

Abbreviations: HDL‐C, high‐density lipoprotein cholesterol; iPTH, intact parathyroid hormone; LDL, low‐density lipoprotein.

**Table 2:** Clinical, biochemical characteristics, and MMSE scores of different groups

Variable	Q1(*n* = 45)	Q2(*n* = 45)	Q3(*n* = 45)	Q4(*n* = 45)	*p*
SUA (μmol/L)	<356.1	356.1–432	432–501.8	>501.8	–
Age (years)	69.1 ± 5.4	70.5 ± 6.1	68.9 ± 5.2	70.8 ± 5.5	.186
Male (*n*)	25	27	25	26	.646
Education (years)	7.74 ± 1.7	7.53 ± 1.4	7.68 ± 1.9	7.62 ± 1.6	.716
Smokers (*n*)	18	23	20	19	.346
systolic BP	140.3 ± 10.2	145.1 ± 9.1	139.6 ± 11.4	143.7 ± 10.8	.503
diastolic BP	79.7 ± 7.1	81.0 ± 6.5	78.7 ± 6.2	80.3 ± 6.8	.760
Duration of dialysis (months)	52.0 ± 7.4	58.3 ± 8.8	53.2 ± 7.6	66.7 ± 8.1	.157
Hemoglobin, g/L	110.2 ± 21.3	115.8 ± 22.4	114.6 ± 17.7	111.6 ± 24.3	.283
Albumin, g/L	38.0 ± 4.1	38.1 ± 4.2	39.4 ± 3.0	38.9 ± 3.5	.349
Blood urea nitrogen, mmol/L	16.1 ± 4.9	24.7 ± 5.2	20.6 ± 3.1	23.9 ± 6.0	.581
Creatinine, μmol/L	485.8 ± 58.8	456.3 ± 60.2	425.9 ± 67.1	472.4 ± 52.1	.155
Uric acid, μmol/L	273.1 ± 26.7	393.6 ± 24.3	466.6 ± 21.7	568.9 ± 26.5	.435
Sodium, mmol/L	139.3 ± 4.8	137.4 ± 6.9	139.9 ± 5.7	138.1 ± 6.5	.309
Potassium, mmol/L	4.5 ± 0.9	4.7 ± 0.9	4.9 ± 0.8	4.9 ± 0.4	.542
Calcium, mmol/L	1.8 ± 0.2	1.8 ± 0.1	1.8 ± 0.3	1.8 ± 0.2	.222
Phosphorus, mmol/L	1.6 ± 0.3	1.6 ± 0.4	1.9 ± 0.3	1.8 ± 0.2	.652
Blood glucose, mmol/L	5.6 ± 1.6	6.6 ± 1.4	6.3 ± 2.1	6.4 ± 2.0	.433
Total cholesterol, mmol/L	3.7 ± 1.0	3.5 ± 1.1	3.7 ± 0.9	3.8 ± 0.8	.692
Triglyceride, mmol/L	1.2 ± 0.4	1.4 ± 0.5	1.8 ± 0.3	1.6 ± 0.4	.253
HDL‐C, mmol/L	1.1 ± 0.3	1.2 ± 0.3	1.2 ± 0.3	1.1 ± 0.2	.224
LDL‐C, mmol/L	1.9 ± 0.3	1.8 ± 0.5	2.1 ± 0.2	1.8 ± 0.3	.279
iPTH, pg/ml	396.1 ± 23.8	414.9 ± 22.2	384.5 ± 25.9	388.3 ± 24.5	.248
MMSE score	28.1 ± 0.9	26.2 ± 1.1	24.1 ± 0.8	21.9 ± 0.9	.006

Abbreviations: HDL‐C, high‐density lipoprotein cholesterol; iPTH, intact parathyroid hormone; LDL, low‐density lipoprotein.

In the Spearman rank correlation test, MMSE score showed negative correlation with SUA (*r* = −0.307, *p* = .014) and other parameters, such as age, years of education, serum concentrations of hemoglobin, total cholesterol, and iPTH, which also had significant correlation with MMSE score (*p* < .05, Table 3).

**Table 3:** Correlation analysis between MMSE and parameters

	MMSE	
	*r*	*p*
Age (years)	.246	.027
Male (*n*)	.120	.471
Education (years)	.306	.014
Smokers (*n*)	.369	.613
Systolic BP	.261	.635
Diastolic BP	.218	.772
Duration of dialysis (months)	.296	.153
Hemoglobin, g/L	.266	.037
Albumin, g/L	.311	.282
Blood urea nitrogen, mmol/L	.250	.781
Creatinine, μmol/L	.135	.643
Sodium, mmol/L	.254	.836
Potassium, mmol/L	.402	.517
Calcium, mmol/L	.199	.623
Phosphorus, mmol/L	.340	.288
Blood glucose, mmol/L	.201	.146
Total cholesterol, mmol/L	.339	.031
Triglyceride, mmol/L	.128	.718
HDL‐C, mmol/L	.272	.592
LDL‐C, mmol/L	.186	.207
iPTH, pg/ml	.313	.019
Uric acid, μmol/L	.307	.014

Abbreviations: HDL‐C, high‐density lipoprotein cholesterol; iPTH, intact parathyroid hormone; LDL, low‐density lipoprotein.

The logistic regression analysis (Table 3) shows association between MMSE scores and parameters in the elderly MHD patients. After controlling of gender, age, smoking habit, duration of dialysis, and the clinical characteristics, compared with the subjects in quartile 1 (SUA < 356.1 μmol/L), there was significant increased risk of CI with the subjects in quartile 4 (SUA > 501.8 μmol/L) (OR = 2.381; 95% CI 0.196–0.375; *p* = .013). The subjects in quartile 2 (SUA: 356.1–432 μmol/L) also had a tendency for the reduced MMSE score compared with those in quartile 1 (OR = 1.672), but there was no significant difference between quartile 1 and quartile 2 (*p* = .089) (Table 4).

**Table 4:** Association between SUA and MMSE scores

	OR(95%CI), *p* Value			
	Q1	Q2	Q3	Q4
*N*	45	45	45	45
MMSE	1	1.672 (0.735–1.221), 0.089	2.074 (0.470–0.773), 0.024	2.381 (0.196–0.375), 0.013

## 4 DISCUSSION

In this study, we found that increased SUA level is negatively associated with the MMSE score in the elderly patients receiving MHD. This association was independent of the effects of age, gender, smoking habit, duration of dialysis, SBP, DBP, and clinical characteristics. This study also showed that SUA was an independent risk factor for CI in the elderly MHD patients.

It was reported that 16 ~ 38% of ESRD patients have CI including dementia, approximately threefold higher than the value observed in age‐matched controls (Kurella Tamura & Yaffe, 2011). In Murray's study (Murray et al., 2006), 37% of 338 HD patients over the age of 55 suffered from severe CI. Fadili et al (Fadili et al., 2014) found that 25% of HD patients presented as a MMSE score of 24 or less. In addition, more than 70% of HD patients older or equal to 55 years have moderate‐to‐severe CI (Murray, 2008). However, in our study, only 15% HD patients over 60 years old appeared CI, which is close to the Japanese study (Odagiri et al., 2011) that reported the prevalence rate of 18.8% among 154 Japanese dialysis patients. The causes may be lie in two factors: Firstly, the sample size is similar but limited; secondly, the subjects of two studies were all Asians.

In Pei's study (Pei et al., 2019), the Spearman correlation test indicated that age, education years, uric acid, serum albumin, and blood pressure were related to CI in HD patients, while in our study, the logistic regression analysis indicated only age, years of education, and SUA were related to CI in MHD population except general risk factors such as hemoglobin and iPTH. This may be due to different age of subjects in two studies. In our study, our patients were all over 60 years old. In addition, most of our patients have stable blood pressure, which has no significant difference among four groups.

Several studies suggested that CI of the general population might be related to traditional vascular risk factors including hypertension, diabetes, age, smoking, and dyslipidemia as well as nontraditional risk factors such as hyperhomocysteinemia, inflammation, and oxidative stress (DeCarli, 2003). As for ESRD patients, anemia and serum albumin and parathyroid hormone level had been demonstrated to be associated with CI (Herrmann, Safran, Levkoff, & Minaker, 1992; Stivelman, 2000), which is similar to our study. However, there was no significant difference in the concentration of serum albumin among the SUA quartiles in our study, which is consistent with Sun Hwa Lee's study (Lee, Cho, Min, Lee, & Jung, 2018).

In addition to the above‐mentioned insults, the effect of uremic toxins on neurons also resulted in CI in CKD patients (Johnston et al., 2004). While as for HD population, CI could also be initiated by hemodynamic instability during dialysis, hemodialysis volume, inflammation, oxidative stress, anemia, or malnutrition (Kalaitzidis et al., 2013; Kurella, Mapes, Port, & Chertow, 2006; Murray et al., 2007; Prohovnik et al., 2007; Yoshimitsu et al., 2000).

In this study, patients with higher SUA quartile had significantly lower MMSE scores (*p* < .001). There is negative correlation between the SUA level and MMSE scores in the Spearman correlation test (*r* = −.307; *p* < .05). A cohort study reported that increased uric acid was associated with poorer working memory in cognitively healthy community‐dwelling older women (Vannorsdall, Kueider, Carlson, & Schretlen, 2014). And hyperuricemia is related to white matter atrophy, worse cognition (Verhaaren et al., 2013), and cerebral ischemic burden (Schretlen et al., 2007). What's more, it is also associated with faster cognitive function decline in visual memory and visuo‐construction ability, although increased serum uric acid overtime was associated with a beneficial effect for the attention and processing speed among older men (Beydoun et al., 2016).

Therefore, there is a controversy about uric acid. Some studies reported that higher level of SUA was associated with poorer cognitive performance (Cervellati et al., 2014; Cicero et al., 2015), while others suggested a beneficial effect (Al‐khateeb et al., 2015; Beydoun et al., 2016), which may be associated with uric acid antioxidant (primarily in plasma) and oxidant (primarily intracellular) function in neurons (Sautin & Johnson, 2008). On the one hand, uric acid could stimulate oxidative stress (Corry et al., 2008). In addition, HD per se may stimulate an extra oxidative stress, furtherly attributed to antioxidants removed through dialyzer, and initiate neutrophil NADPH oxidase inflammative reaction to produce reactive oxygen species (Glorieux, Neirynck, Veys, & Vanholder, 2012; Modaresi, Nafar, & Sahraei, 2015). Furthermore, routine intravenous iron supplementation could further aggravate oxidative stress in MHD patients (Drueke et al., 2002). On the other hand, it was reported that uric acid itself has antioxidant properties, which may reduce the burden of cellular oxidation, a major cause of neurodegenerative diseases (Sautin & Johnson, 2008).

In our study, we found that the patients in Q4 group (SUA > 501.8 μmol/L) had lowest MMSE scores. However, on the contrary, previous research showed that SUA level was lower in those with mild cognitive impairment (MCI), Alzheimer's disease (AD), and vascular dementia when compared with cognitively healthy controls (Khan, Quinn, Hewitt, Fan, & Dawson, 2016; Xu et al., 2017). It may be associated with different spectrum of diseases.

In conclusion, hyperuricemia affects CI in MHD patients over 60 years old. The underlying mechanism awaits to explore further.

### 4.1 Limitations of the study

There were some limitations in our study. Firstly, relatively few number of patients was under study, in which the possibility of selection bias could not be excluded. Thus, it is recommended to carry out studies with larger sample sizes, and we believe that enlarging the sample numbers would help to ascertain whether elevated UA raises the incidence of CI in the MHD patients. Secondly, this study includes a certain number of MHD patients over 60 years old. Therefore, the findings could not account for all the MHD patients. Thirdly, the parameters about oxidative stress, including MDA, SOD, and CAT, were not included in this study. In the future, we will further test the level of oxidative stress‐associated markers.

## CONFLICT OF INTEREST

The authors have declared no conflict of interest.

## Data Availability

The data that support the findings of this study are available on request from the corresponding author. The data are not publicly available due to privacy or ethical restrictions.
